# Metal-electrode-free Window-like Organic Solar Cells with p-Doped Carbon Nanotube Thin-film Electrodes

**DOI:** 10.1038/srep31348

**Published:** 2016-08-16

**Authors:** Il Jeon, Clement Delacou, Antti Kaskela, Esko I. Kauppinen, Shigeo Maruyama, Yutaka Matsuo

**Affiliations:** 1 Department of Chemistry, School of Science, The University of Tokyo, 7-3-1 Hongo, Bunkyo-ku, Tokyo 113-0033, Japan; 2 Department of Mechanical Engineering, School of Engineering, The University of Tokyo, 7-3-1 Hongo, Bunkyo-ku, Tokyo 113-8656, Japan; 3 Department of Applied Physics, Aalto University School of Science, 15100, FI-00076 Aalto, Finland; 4 National Institute of Advanced Industrial Science and Technology (AIST), 1-2-1 Namiki, Tsukuba, Ibaraki 305-8564, Japan; 5 University of Science and Technology of China, Hefei, Anhui 230026, China

## Abstract

Organic solar cells are flexible and inexpensive, and expected to have a wide range of applications. Many transparent organic solar cells have been reported and their success hinges on full transparency and high power conversion efficiency. Recently, carbon nanotubes and graphene, which meet these criteria, have been used in transparent conductive electrodes. However, their use in top electrodes has been limited by mechanical difficulties in fabrication and doping. Here, expensive metal top electrodes were replaced with high-performance, easy-to-transfer, aerosol-synthesized carbon nanotubes to produce transparent organic solar cells. The carbon nanotubes were p-doped by two new methods: HNO_3_ doping via ‘sandwich transfer’, and MoO_x_ thermal doping via ‘bridge transfer’. Although both of the doping methods improved the performance of the carbon nanotubes and the photovoltaic performance of devices, sandwich transfer, which gave a 4.1% power conversion efficiency, was slightly more effective than bridge transfer, which produced a power conversion efficiency of 3.4%. Applying a thinner carbon nanotube film with 90% transparency decreased the efficiency to 3.7%, which was still high. Overall, the transparent solar cells had an efficiency of around 50% that of non-transparent metal-based solar cells (7.8%).

Organic solar cells (OSCs) have attracted much attention compared with other types of solar cell owing to their low cost, high efficiency, and diverse applications^1^,^2^,^3^. Currently, the power conversion efficiency (PCE) of OSCs has reached around 10% for both tandem and non-tandem architectures, demonstrating that OSCs are promising as solar energy harvesters^4^,^5^. In addition to high efficiency, other properties of OSCs have been intensively investigated^6^,^7^. OSCs are regarded as a green technology that can be wearable, surface conforming, and suitable for windows. For these applications, OSCs must use metal-free, mechanically resilient, and translucent materials, while retaining a high PCE. The first step toward this achievement is replacing the metal electrode, which is expensive and produces bright glare. Previously, many attempts have been made to develop transparent, flexible solar cells for applications such as building-integrated photovoltaics and solar chargers for portable electronics using metallic grids, nanowire networks, metal oxides, or conducting polymers^8^,^9^,^10^,^11^,^12^,^13^,^14^,^15^,^16^,^17^,^18^,^19^,^20^. However, transparent conductors often result in low visible light transparency, low PCEs, or low flexibility, because the material used in device design and fabrication is not suitably transparent and conductive.

Single-walled carbon nanotubes (SWNTs) are expected to address current problems because they are mechanically flexible, made with cheap and abundant carbon, easy to synthesize, and suited to direct roll-to-roll processes^21^. SWNTs are structurally the simplest class of carbon nanotubes with diameters in the range of 0.4–3.0 nm^22^. Following their discovery by Iijima in the early 1990s, their development has continued and now high-quality freestanding pure SWNTs show a transparency of over 90% with a resistance of around 85 Ω/sq^23^. Conductive SWNT films can be used as an electrode to replace indium tin oxide (ITO) in photovoltaics^24^,^25^. However, there are few reports on SWNT films as a top electrode because SWNT lamination is difficult from above^26^,^27^,^28^. Li *et al*.^26^ used SWNT films as the top electrode in perovskite solar cells. However, their SWNT films could not be doped, because it is difficult to dope top-laminated SWNT electrodes without damaging the device.

Here, we report SWNT-based metal-free OSCs with window-like transparency. The SWNT films were p-doped with two dopants, HNO_3_ via sandwich transfer and MoO_3_ via bridge transfer. The HNO_3_-doped and MoO_3_-doped 60% transparent SWNT-laminated OSCs showed PCEs of 4.1% and 3.4%, respectively. Using 90% transparent SWNT films, which produced OSCs visually more similar to a window, resulted in PCEs of 3.7% and 3.1% for HNO_3_-doped and MoO_3_-doped, respectively, whereas the reference ITO-based OSC showed a PCE of 7.8%. Our transparent OSCs, which are suitable for window applications, were fabricated by doping through direct lamination and dry lamination of SWNT films for the top electrode (Fig. 1). The double-sided light response of these transparent yet highly efficient solar cells offers advantages in many applications. We expect that the methods presented here will pave the way to future multifunctional OSCs.

## Results and Discussion

### Aerosol single-walled carbon nanotubes

Randomly oriented SWNT networks with high purity and long nanotube bundle lengths were synthesized by the aerosol chemical vapor deposition (CVD) method^23^,^29^. Floating catalyst aerosol CVD was performed in a scaled-up reaction tube with a diameter of 150 mm. The dry-deposited SWNT networks showed high purity, as confirmed by clear Van Hove peaks in the UV-vis spectra and the low intensity of the defect-derived D band in Raman spectra^30^. Furthermore, because the process required no sonication-based dispersion step, the resulting SWNT network consisted of exceptionally long SWNTs. Facile transferability is another advantage of the aerosol SWNT films. Once deposited from the aerosol, the CNTs showed strong tube-to-tube interactions and assembled into a freestanding thin film. The SWNT films were easily peeled off from a nitrocellulose film with a pair of tweezers and transferred onto other substrates for device fabrication.

### Architectures of the solar cell devices

**Figure 2:**
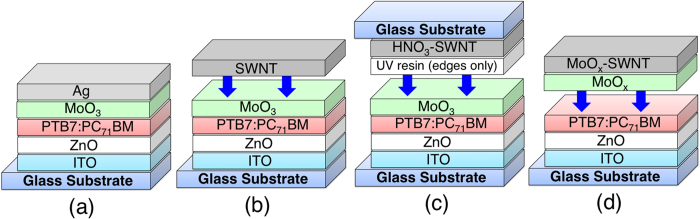
Schematics of the architecture of (**a**) a conventional inverted OSC, (**b**) an SWNT-based transparent OSC, (**c**) a HNO_3_-doped SWNT-based transparent OSC, and (**d**) a MoO_x_-doped SWNT-based transparent OSC.

The structures of SWNT-based transparent OSCs are shown in Fig. 2. Figure 2a shows the conventional inverted OSC structure where Ag was used as an anode. This entails metal deposition and produces a non-transparent device. Figure 2b shows the same structure, except that Ag has been replaced with a highly transparent aerosol SWNT film. The light can be shone from either the ITO or SWNT side, or both sides, to generate photo-induced power. Thus, we refer to it as a window-like transparent OSC. The conductivity and transparency of SWNTs must be enhanced by doping to produce efficient solar cells. Figure 2c,d respectively show HNO_3_-doped and MoO_x_-doped SWNT OSCs with window-like transparency. Owing to the difficulty of doping, two different doping methods were used, resulting in different architectures. A mixture of the low bandgap polymer thieno[3,4-b]thiophene/benzodithiophene (PTB7) and the acceptor [6,6]-phenyl C71-butyric acid methyl ester (PC_71_BM) with an additive, 1,8-diiodoctane (DIO), was used as the photoactive layer in all the devices.

### Performance of SWNT-laminated transparent organic solar cells

Photoluminescence quenching can be used to measure charge extraction ability^31^. A PTB7-based organic photoactive layer was deposited on a glass substrate and an SWNT film was top-laminated. As shown in Figure S1, the spectrum of the organic photoactive layer was suppressed substantially when the SWNT film was placed on top of it. This indicates effective charge extraction and successful lamination.

OSCs were fabricated with 90% transparent SWNT films. The PCEs measured with light shining from the SWNT side, the ITO side, and the ITO side with a mirror reflecting from behind were different (Table 1: Devices B and C). When light was shone from the ITO side, a PCE of 2% was obtained, which was approximately twice the PCE when light was shone from the SWNT side (0.9%). These values were very low compared with the non-transparent conventional reference, Device A (7.8%), because the SWNT films were not doped. The UV-vis spectra (Figure S2a) showed that ITO was more transparent than the 90% transparent SWNT film. The difference increased when we included the whole device, including the photoactive layer, ZnO, and MoO_3_. The increased difference in transmittance is surmised to be due to the internal surface reflection between the layers. When adjacent two layers have difference in refractive index, internal surface reflection occurs. Thus, the greater the difference, the greater the transmittance loss. In other words, shining light on the ITO side [glass(1.5)/ITO(1.8)/ZnO(2.0)/PTB7:PCBM(1.6)] optically advantageous for solar cell performance compared to the SWNT side [glass(1.5)/SWNT(2.5)/MoO_3_(2.2)/PTB7:PCBM(1.6)]. This results in a higher PCE for Device C than for Device B because a larger number of photons are converted to a higher short-circuit current density (*J*_SC_) in Device C. The incident photon-to-current efficiency (IPCE) was measured to confirm this. As expected, when the light was shone from the ITO side, more charges were extracted (Figure S2b). The same behavior was observed from the doped SWNT-based devices as well.

Compared with Device B, Device C showed not only a higher *J*_SC_ but also a higher open-circuit voltage (*V*_OC_) and fill factor (FF). This is a typical characteristic of solar cells that can be described by the Shockley equation. In principle, it is related to logarithmic scaling of *V*_OC_ with light intensity^32^. Therefore, Device C with a higher *J*_SC_ will exhibit a higher *V*_OC_. Equation (1) shows that FF is also affected by *V*_OC_^33^. This is especially true in real solar cell devices, which show non-ideal diode behavior. Thus, low *J*_SC_ can induce low *V*_OC_ and FF.
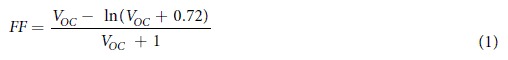


**Figure 3:**
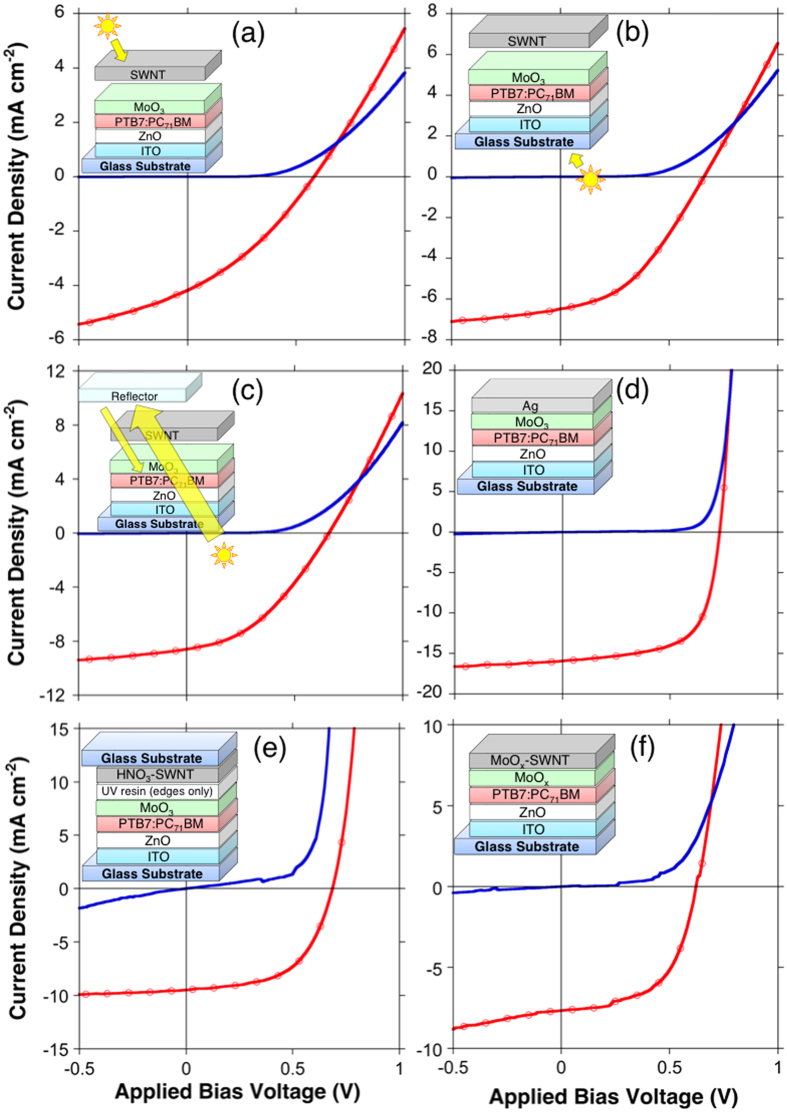
*J-V* curves under one sun (red dotted) and in the dark (blue plane) for (**a**) an SWNT-based transparent OSC with light from the SWNT side, (**b**) an SWNT-based transparent OSC with light from the ITO side, (**c**) an SWNT-based transparent OSC with light from the ITO side and a reflector, (**d**) a conventional inverted OSC, (**e**) a HNO_3_-SWNT sandwich transfer OSC with light from the ITO side, and (**f**) a MoO_x_-SWNT bridge transfer OSC with light from the ITO side.

The shunt resistance (*R*_SH_) is particularly important in transparent OSCs because the light intensity is not sufficient. At low light intensity, both the bias point and the current of the solar cell devices decrease. This causes the equivalent resistance of the solar cell devices to approach *R*_SH_^34^. If the equivalent and shunt resistances are similar, the fraction of the total current flowing through the *R*_SH_ will increase, and this may lead to the recombination of charges. Therefore, it is crucial that we have a device system with a sufficiently high *R*_SH_ value to avoid recombination. The current-voltage (*J-V*) curves in Fig. 3a,b show that 90% transparent SWNT OSCs possess sufficiently high *R*_SH_ regardless of the light direction.

In conventional OSCs, metal electrodes can act as a rear reflector to direct unabsorbed light back to the photoactive layer. This provides a higher photocurrent, especially in the wavelength region below 700 nm. However, for the transparent OSCs, because no light can be reflected back and the active material is not thick enough to absorb all the sunlight, much light passes through unabsorbed. When a silver reflector (mirror) was placed on the opposite side of the light source, the *J*_SC_ increased from 6.5 to 8.6 mA/cm^2^ (Table 1: Device D; Fig. 3d). However, despite the increased light intensity, *V*_OC_ and FF did not increase further. This reveals that the maximum *V*_OC_ obtained by using pristine 90% transparent SWNTs is limited to around 0.66 V. This could be due to imperfect interface contact between SWNTs and MoO_3_^35^,^36^ as demonstrated by the unparalleled dark *J-V* curves and light *J-V* curves at high current density. In general, *V*_OC_ is controlled by the difference between the highest occupied molecular orbital (HOMO) of a donor and the lowest unoccupied molecular orbital (LUMO) of an acceptor. Furthermore, the HOMO and LUMO are affected by the interfacial layers’ Fermi levels and the electrodes’ work functions^37^. Therefore, poor contact between the SWNTs and MoO_3_ may have been the limiting factor for the *V*_OC_. The overall PCE improvement was only 0.4%. This suggests that the double-sided light response of the transparent OSCs leads to sufficient photon excitations and that using a reflector at the cost of losing the transparency is not desirable.

### Doping methodologies for SWNT-laminated transparent solar cells

Although transparent OSCs were achieved as shown above, SWNTs should be p-doped to improve the conductivity and transmittance to boost the PCE of the OSCs. Doping top-laminated SWNTs has not been reported because of the mechanical difficulty of doping. Unlike SWNTs on a glass substrate, doping top-laminated SWNTs damages the device underneath. Hence, in this work, we devised two methods for safely doping SWNTs with HNO_3_ or MoO_3_.

**Figure 4:**
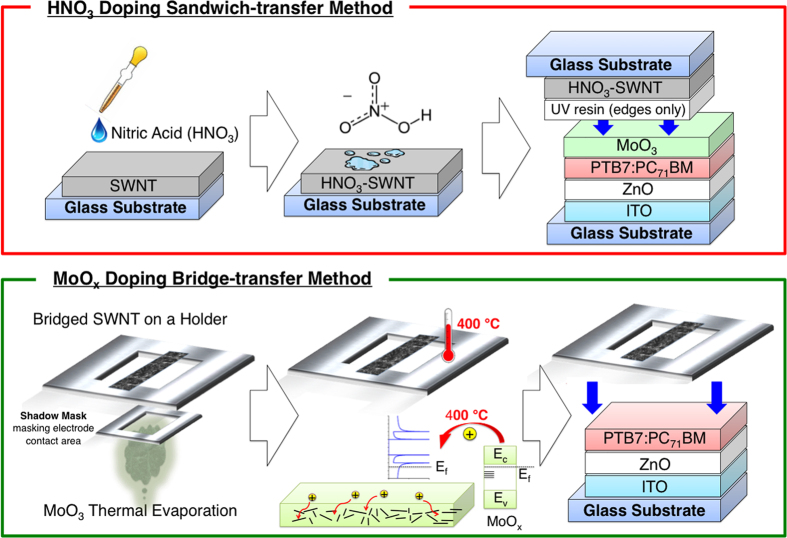
Schematics of HNO_3_ doping sandwich transfer process (above) and MoO_x_ thermal doping bridge transfer process (below).

HNO_3_(aq) acid is an effective p-dopant^38^. Nevertheless, its high acidity makes it impossible to apply directly. When a drop of HNO_3_ was applied to an SWNT laminated device, it percolated through the film and completely destroyed the organic materials underneath (Figure S3). Therefore, doping was performed on the SWNT film separately first. Figure 4a shows how the HNO_3_ sandwich transfer was performed. One drop of HNO_3_ was applied to an SWNT film on a glass substrate followed by heating at 80 °C for 5 min. The SWNT film turned slightly reddish as the acid dried and this signified successful doping. A decrease in the Fermi level value from −5.0 to −6.0 eV by photoelectron yield spectroscopy (PYS) confirmed a successful p-doping. The HNO_3_-SWNT film was sandwiched onto a MoO_3_ film on a partially fabricated OSC. UV resin was applied at the edges to reinforce the adhesion. A PCE of 3.7% was achieved with the light source positioned at the ITO side (Table 1: Device E). An increase in *J*_SC_ and a reduction in series resistance (*R*_S_) confirmed the improvement of the transparency and conductivity of the HNO_3_-doped SWNT OSC. The increase in *V*_OC_ meant that the interfacial contact improved. We suggest this is because of the pressure applied to HNO_3_-SWNT OSC during the sandwich transfer.

Thermal MoO_x_ doping of SWNTs is a more stable doping method than doping with HNO_3_ despite its slightly lower effectiveness^39^. The method has been used in OSCs^40^, but it is not suitable for top-laminated SWNT films, because it requires high-temperature annealing above 300 °C. Thus, we propose a bridge transfer method (Fig. 4b). An SWNT film was transferred onto a metal holder where the film was hung like a bridge (Figure S4). A shadow mask was placed below the SWNT film to mask the electrode contact area. MoO_3_ was deposited from below by vacuum thermal evaporation. The MoO_3_-SWNT film was then annealed at 400 °C together with the holder to boost the doping effect and reduce the film to MoO_x_-SWNT, where x is between 2 and 3^39^. A decrease in the Fermi level value from −5.0 to −5.6 eV by photoelectron yield spectroscopy (PYS) confirmed a successful p-doping. MoO_x_-SWNT was gently laminated by using the holder on a partially fabricated device, where the MoO_3_ film was not deposited because the MoO_x_ on the SWNTs functioned as both a dopant and electron-transporting layer. A PCE of 3.1% was recorded for this device (Table 1: Device F). *J*_SC_ was lower and *R*_S_ was higher than those of Device E because MoO_x_ thermal doping was less effective than HNO_3_ doping. Importantly, because the SWNT film was hung precariously on the metal holder, extra caution was necessary during handling. Any small external impact or draft strong enough to crumple the SWNT film created microwrinkles, which were invisible to the naked eye, but were detected by atomic force microscopy (AFM; Figure S5). The lower *V*_OC_ in this device may have been caused by the remnants of microwrinkles undermining the interface. Furthermore, pressure was not applied during the lamination of the SWNT film unlike the sandwich transfer method. Compared with the HNO_3_-SWNT sandwich transfer, the bridge transfer method had lower reproducibility because it is a sensitive process.

Despite high PCEs, both doping methods suffered from instability in the *J-V* sweeps (Figure 3e,f). We ascribe this to the mechanical variability of the fabrication methods, namely excess pressure applied to the SWNT film during the HNO_3_-SWNT sandwich transfer, and the sensitivity of the MoO_x_-SWNT bridge transfer method. However, if the processes are mechanically optimized, high efficiency and stability could be obtained.

### Application of thicker SWNT films

**Figure 5:**
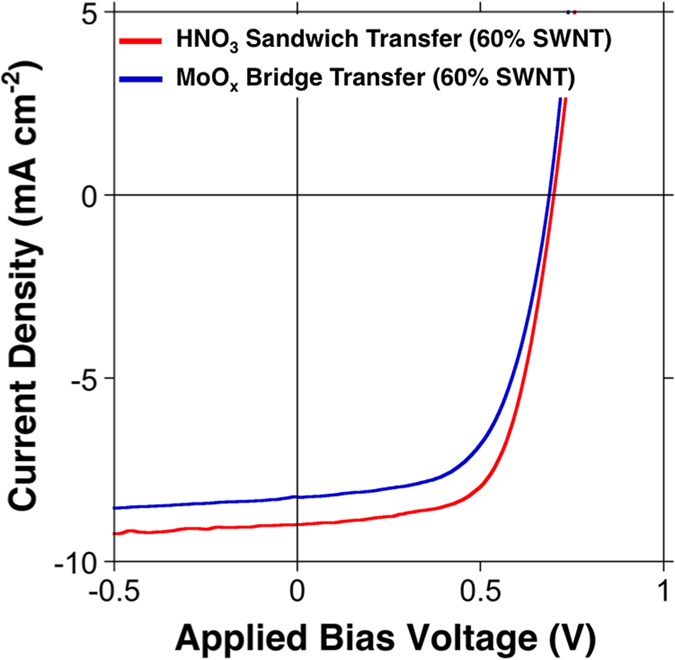
*J-V* curves of 60% transparent SWNT-based OSCs fabricated by the HNO_3_ sandwich transfer method (red line) and the MoOx bridge transfer method (blue line).

Thicker SWNT films possess higher conductivity, although their transmittance is lower. By incorporating the thicker SWNT films (60% transparency at 550 nm wavelength), higher PCEs were obtained (Figures 5 and S6). The PCE of the HNO_3_-doped device was 4.1% (Table 1: Device G) and that of the MoO_x_-doped device was 3.4% (Table 1: Device H). Because of the higher conductivity of the 60% transparent SWNT films, the FF was higher than that of the 90% transparent SWNT-based devices by around 0.1. Interestingly, *V*_OC_ of Device H was higher than expected. We attribute this to thicker SWNT films being less vulnerable to microwrinkle formation during the bridge transfer. Despite the lower transmittance of the films, Devices G and H displayed high *J*_SC_, because the main source of photons came from the ITO side not the SWNT side. Although it may seem obvious to use a thicker SWNT film to gain higher PCEs, it would compromise the transparency of the OSCs (Figure S7). The improvement in PCE could be achieved only at the expense of the transparency.

## Conclusions

In conclusion, undoped SWNT (90% transmittance)-based MoO_3_/PTB7:PC_71_BM:DIO/ZnO/ITO transparent OSCs showed a PCE of 1.8%. The aerosol-synthesized SWNT electrode, which was laminated from above as a top electrode, was easy to fabricate, chemically stable, electrically compatible, and mechanically resilient. Applying p-doping to the SWNT film through our novel HNO_3_ sandwich transfer and MoO_3_ bridge transfer methods, the PCEs of the transparent OSCs increased to 3.7% and 3.1%, respectively. An even higher PCE of 4.1% was obtained at the expense of transparency by incorporating thicker SWNT films. By replacing the metal electrodes, these OSCs were inexpensive, had window-like transparency, and were visually glare-free. This research demonstrated the promising potential in window solar cell applications and flexible tandem OSCs.

## Experimental Methods

### Aerosol SWNT Preparation

SWNTs were synthesized by an aerosol (floating catalyst) CVD method based on ferrocene vapor decomposition in a CO atmosphere. The catalyst precursor was vaporized by passing room-temperature temperature CO through a cartridge filled with ferrocene powder. The flow containing the ferrocene vapor was then introduced into the high-temperature zone of a ceramic tube reactor through a water-cooled probe and mixed with additional CO. To obtain stable SWNT growth, a controlled amount of CO_2_ was added together with the carbon source (CO). SWNTs were directly collected downstream of the reactor by filtering the flow through a nitrocellulose or silver membrane filter (HAWP, Millipore Corp., USA; 0.45 μm pore diameter).

### Device Fabrication

For the reference device, ITO substrates 15 × 15 mm in size with an active area of 3 × 3 mm and a sheet resistance of 9 Ω/square (Techno Print Co., Ltd.) were sonicated in cleaning surfactant (Semi Clean, M-Lo), water, acetone, and 2-isopropanol for 15 min each. The substrates were then dried with a nitrogen gun. ITO substrates were exposed to UV/O_3_ for 30 min to remove any remaining organic impurities. They were transferred to a nitrogen-filled glovebox for further fabrication. ZnO sol–gel films were prepared by the method reported by Heeger *et al*.^41^. The metal oxides were baked at 200 °C before depositing the photoactive layer.

For the photoactive layer deposition, PTB7 and PC_71_BM (Luminescence Technology Corp.) were used as received. A solution of PTB7 and PC_71_BM was prepared in a mixed solvent of chlorobenzene (CB; 99%) and DIO (97:3). PTB7 (10 mg) and PC_71_BM (15 mg) were initially dissolved in CB in a nitrogen glovebox (0.97 mL). The solution was stirred overnight at 60 °C. After 24 h, DIO (30 μL) was added and the solution was stirred for 1 h at 70 °C. The PTB7:PC_71_BM:DIO solution (80 nm thick) was spin coated at 1500 rpm for 60 s on a ZnO layer to give a film approximately 100 nm thick. For the hole-transporting layer, a 15-nm-thick MoO_3_ layer was deposited on top, under vacuum via a thermal evaporator at a rate of 0.2 Å/s. To improve the contact between the solar simulator and the SWNT film, an Ag (100 nm) pattern was deposited only at the contacts where the solar simulator wires were placed.

### Transfer of HNO_3_-doped SWNT Films by the Sandwich Transfer Method

The SWNT film was transferred to a bare glass substrate. HNO_3_ (70% in water) was applied dropwise and dried at 80 °C to p-dope the SWNT films. The HNO_3_-doped SWNT substrates were sandwiched onto a MoO_3_ and Ag-patterned device (MoO_3_/PTB7:PC_71_BM:DIO/ZnO/ITO) and UV resin was applied at the edges to hold the two substrates and encapsulate the device.

### Transfer of MoO_x_-doped SWNT Films by the Bridge Transfer Method

A special holder for SWNT films was prepared. An SWNT film was transferred onto the holder so that the film was hung like a bridge. A 15-nm-thick MoO_3_ layer was thermally deposited on the bridged SWNT film followed by thermal annealing at 300 °C for 3 h anaerobically to induce MoO_x_ doping. The bridged SWNT film was transferred carefully to the Ag-patterned photoactive layer. A drop of PEDOT:PSS was applied and it was spin coated at 4500 rpm for 60 s to assist lamination. Because MoO_x_ also functions as the hole-transporting layer, the MoO_3_ step was omitted in this method. In other words, SWNT/MoO_x_ was laminated on to PTB7:PC_71_BM:DIO/ZnO/ITO rather than MoO_3_/PTB7:PC_71_BM:DIO/ZnO/ITO.

### Characterization

*J–V* characteristics were measured by a software-controlled source meter (2400, Keithley) in the dark and under 1 sun AM 1.5G simulated sunlight irradiation (100 mW/cm^2^) by using a solar simulator (EMS-35AAA, Ushio Spax Inc.), which was calibrated with a silicon diode (BS-520BK, Bunkokeiki). Topographic images were recorded by AFM (SPI3800N, SII) operating in tapping mode. The devices were also characterized by scanning electron microscopy (S-4800, Hitachi), Raman microscopy (inVia, Renishaw), and UV-vis-NIR spectroscopy (UV-3150, Shimadzu). Fermi levels were measured by Riken Keiki PYS-A AC-2 and kelvin probe S spectroscopy in air (ESA). They were calibrated by Au before the measurement.

## Additional Information

**How to cite this article**: Jeon, I. *et al*. Metal-electrode-free Window-like Organic Solar Cells with p-Doped Carbon Nanotube Thin-film Electrodes. *Sci. Rep.* **6**, 31348; doi: 10.1038/srep31348 (2016).

## Supplementary Material

Supplementary Information

## Figures and Tables

**Figure 1:**
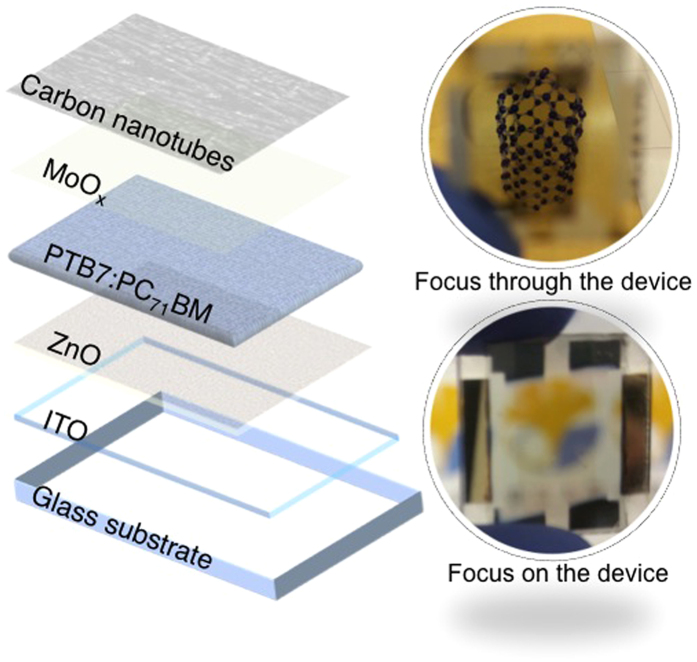
Schematic of the SWNT-laminated transparent solar cell (left) and photographs with different foci (right).

**Table 1:** Photovoltaic performance for SWNT-based transparent inverted OSCs under one sun, AM 1.5G illumination (100 mW/cm^2^).

Device	Light direction	Anode	Dopant	*V*_OC_(V)	*J*_SC_ (mA/cm^2^)	FF	*R*_S_ (Ωcm^2^)	*R*_SH_ (Ωcm^2^)	PCE_best_ (%)
A	from ITO	Ag	None	0.73	16.0	0.65	16	6.4 × 10^4^	7.8
B	from SWNT	SWNT T = 90%		0.58	4.8	0.32	470	4.6 × 10^5^	0.9
C	from ITO			0.66	6.5	0.40	320	8.9 × 10^4^	1.8
D	with reflector			0.66	8.6	0.39	280	5.8 × 10^4^	2.2
E	from ITO	SWNT T = 90%	HNO_3_	0.69	9.5	0.56	70	1.5 × 10^4^	3.7
F			MoO_x_	0.62	8.8	0.56	100	1.8 × 10^5^	3.1
G		SWNT T = 60%	HNO_3_	0.70	9.0	0.65	53	1.6 × 10^7^	4.1
H			MoO_x_	0.68	8.2	0.60	61	8.4 × 10^5^	3.4

Footnote: T = transmittance.
