# Supplementary material for: Metal-electrode-free Window-like Organic Solar Cells with p-Doped Carbon Nanotube Thin-film Electrodes
Source: Sci Rep. 2016 Aug 16;6:31348. doi: 10.1038/srep31348 (PMC4985658; doi:10.1038/srep31348)
Supplement: Supplementary Information [file srep31348-s1.pdf]

Supplementary Information

Metal-electrode-free Window-like Organic Solar Cells with p-Doped Carbon Nanotube Thin-film Electrodes

Il Jeon[1], Clement Delacou[2], Antti Kaskela[3], Esko I. Kauppinen[3], Shigeo Maruyama[2,4], and Yutaka Matsuo[1,2,5]*

1: *Department of Chemistry, School of Science, The University of Tokyo, 7-3-1 Hongo, Bunkyo-ku, Tokyo 113-0033, Japan*

2: *Department of Mechanical Engineering, School of Engineering, The University of Tokyo, 7-3-1 Hongo, Bunkyo-ku, Tokyo 113-8656, Japan*

3: *Department of Applied Physics, Aalto University School of Science, 15100, FI-00076 Aalto, Finland*

4: *National Institute of Advanced Industrial Science and Technology (AIST), 1-2-1 Namiki, Tsukuba, 305-8564, Japan*

5: *University of Science and Technology of China, Hefei, Anhui 230026, China*

Contents:

1. Photoluminescence data

2. UV-vis and IPCE data

3. Direct application of HNO_3(aq) on SWNT-based transparent OSC

4. Bridged SWNT

5. AFM images of micro-wrinkles

6. Cross-sectional SEM images

7. *J-V* curves of 60% transparent SWNT-based transparent OSCs

8. Device pictures

1. Photoluminescence data

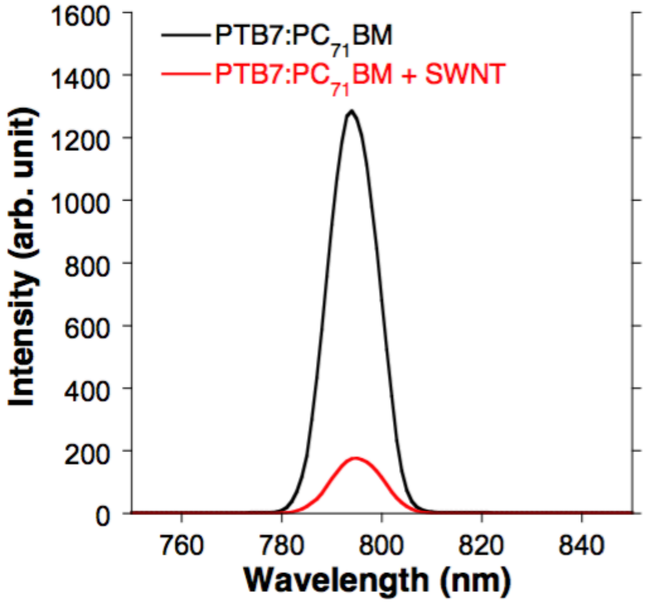

Figure S1. Photoluminescence spectrum of PTB7:$PC_{71}BM$ active layer is subdued when SWNT film is over-coated on the very same active layer.

2. UV-vis and IPCE data

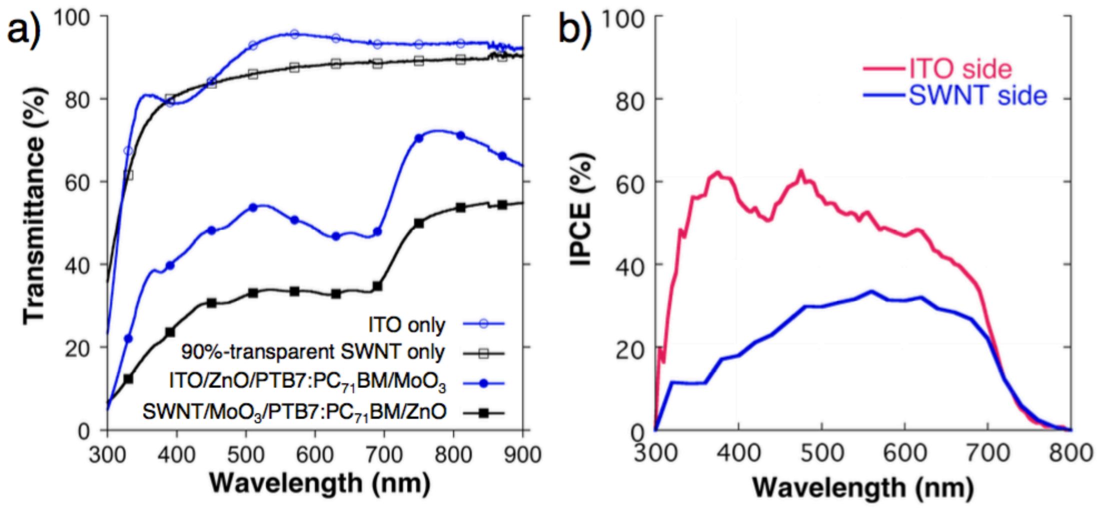

Figure S2. a) UV-vis transmittance measurement spectra of glass/ITO, glass/90%-SWNT, glass/ITO/ZnO/PTB7:$PC_{71}BM/MoO_3$, and glass/90%-SWNT/MoO_3/PTB7:$PC_{71}BM$/ZnO. b) IPCE measurement spectra of transparent OSCs when light is shone from ITO side and when light is shone from SWNT side.

3. Direct application of HNO_3(aq) on SWNT-based transparent OSC

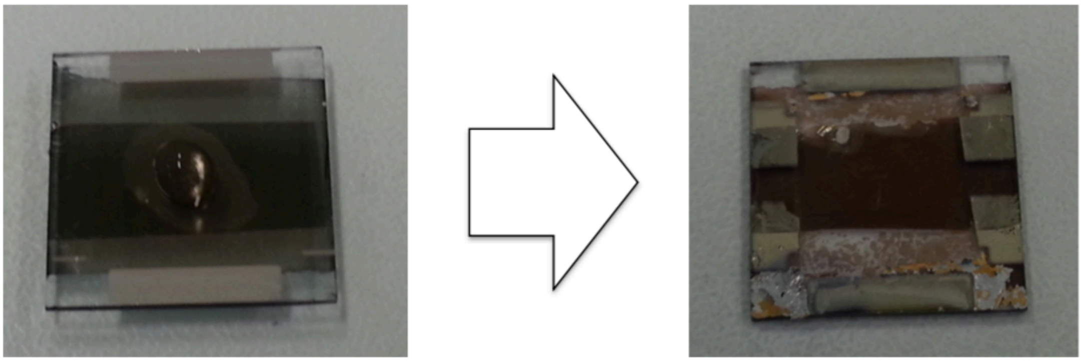

Figure S3. Application of HNO_3(aq) directly onto a SWNT-based transparent OSC (left) will lead to a holistic destruction of the device system (right) within 5 h.

4. Bridged SWNT

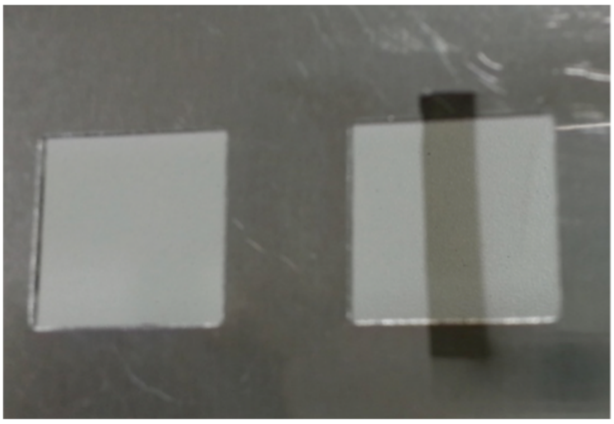

Figure S4. A picture of a SWNT film bridged on a holder.

5. AFM images of micro-wrinkles

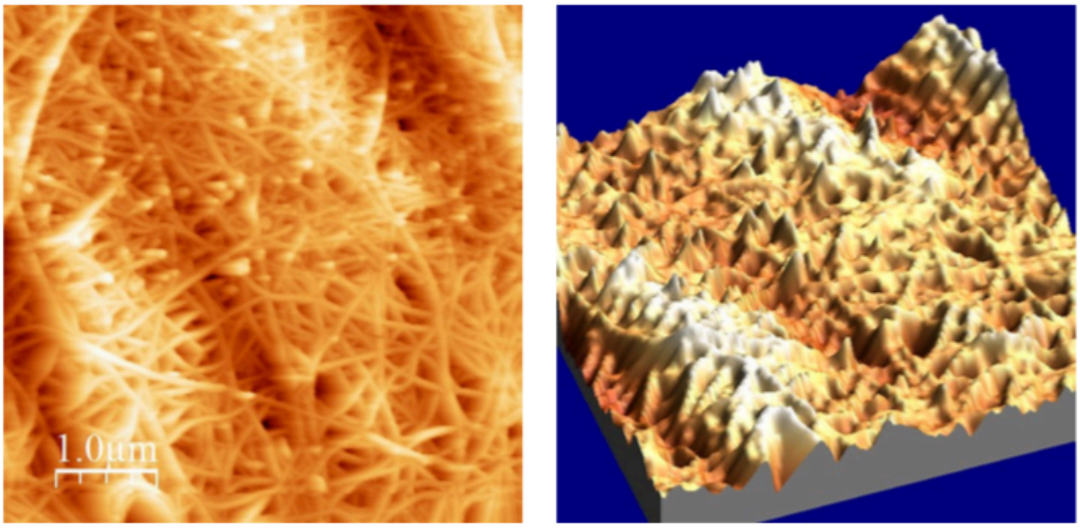

Figure S5. AFM 2D image (left) and 3D image (right) of micro wrinkles formed on bridge-transferred SWNT.

6. Cross-sectional SEM images

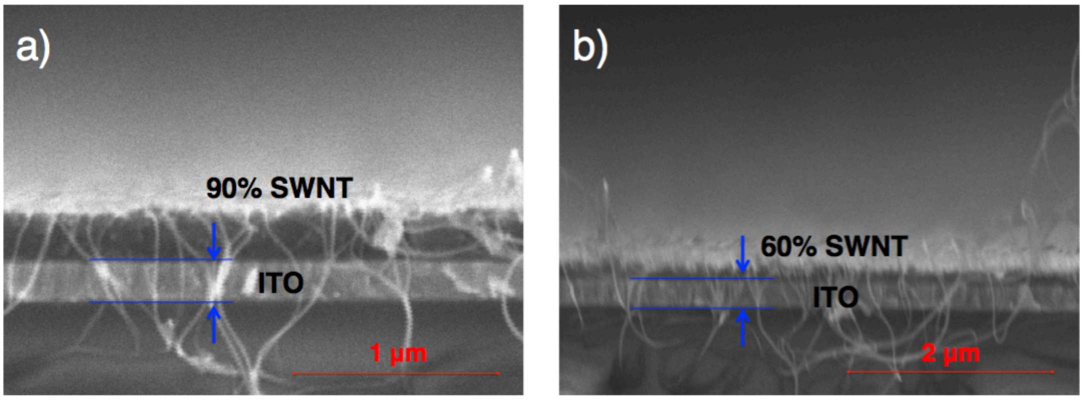

Figure S6. Cross-sectional SEM images of transparent OSC devices with a) a 90%-SWNT film and b) a 60%-SWNT film.

7. Device pictures

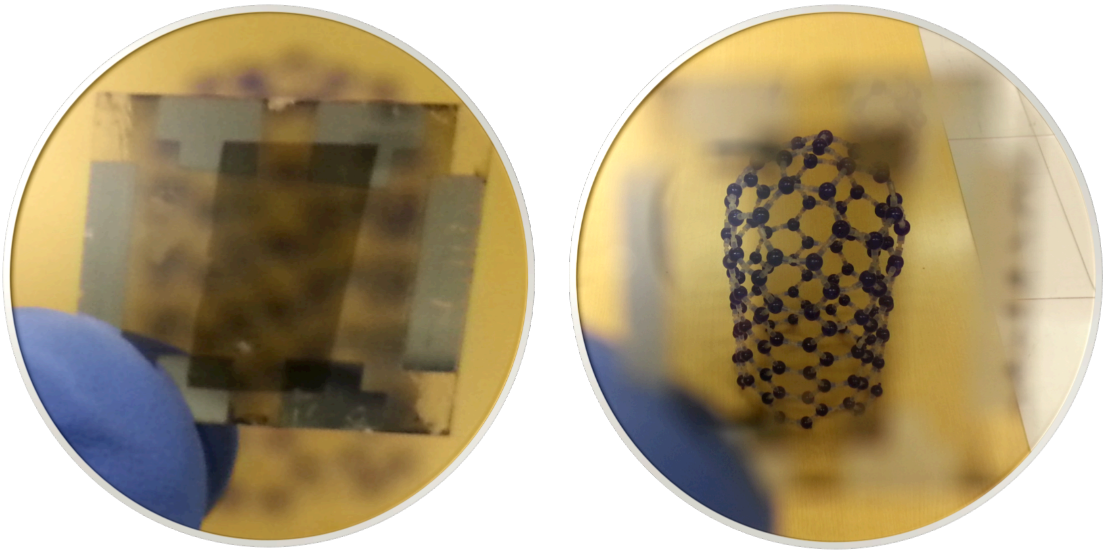

Figure S7. Pictures of 60% transparent SWNT-based transparent OSC when focused on the device (left) and on a behind object, the SWNT plastic model (right).
